# Careful neuropsychological testing reveals a novel genetic marker, GSTO1*C, linked to the pre-stage of Alzheimer's disease

**DOI:** 10.18632/oncotarget.9773

**Published:** 2016-06-01

**Authors:** Ellen Umlauf, Eduard Rappold, Bettina Schiller, Petra Fuchs, Michael Rainer, Brigitte Wolf, Maria Zellner

**Affiliations:** ^1^ Medical University of Vienna, Center of Physiology and Pharmacology, Institute of Physiology, Vienna, Austria; ^2^ SMZ Otto Wagner Spital, 3rd Department of Psychiatry, Vienna, Austria; ^3^ SMZ Ost, Karl Landsteiner Institut für Gedächtnis- und Alzheimerforschung, Vienna, Austria; ^4^ Medical University of Vienna, Surgery Research Laboratory, Department of Surgery, Vienna, Austria

**Keywords:** Alzheimer's disease, MCI, CERAD-Plus, MMSE, well-defined control group, Gerotarget

## Abstract

Approximately 30 million people currently suffer from late-onset Alzheimer's disease (LOAD) worldwide. Twin studies demonstrated that 60 to 80% of LOAD is genetically determined, 20% of which remaining unassigned. This case-control study included 118 cognitively healthy controls, 52 patients with mild cognitive impairment (MCI; the pre-stage of LOAD) and 71 LOAD patients. The participants were genotyped for the genetic LOAD marker apolipoprotein E4 (*APOE4*) and the single-nucleotide polymorphism rs4925 in glutathione S-transferase omega-1 (*GSTO1*). Additive logistic regression showed a novel, statistically significant association of the major allele GSTO1*C with MCI (OR1.9; *p* = 0.032). However, identification of significant SNP-disease relations required well-defined study groups. When classifying participants solely by the short Mini Mental State examination (MMSE), the associations of GSTO1*C and the reference marker *APOE4* with MCI were cancelled. Moreover, even identifying only the control group by MMSE nullified a statistically significant association (OR1.8; *p* = 0.045) between GSTO1*C and LOAD. In contrast, these statistical relations were retained when the detailed Consortium to Establish a Registry for Alzheimer's Disease (CERAD-Plus) test battery was used. Hence, besides proposing rs4925 as a genetic marker for cognitive impairment, this work also emphasized the importance of carefully characterized controls in addition to well-diagnosed patients in case-control studies.

## INTRODUCTION

Late-onset Alzheimer's disease (LOAD), a multifactorial neurodegenerative disorder, is the most prominent form of dementia among the elderly (> 65 years), and its incidence is expected to double within the next 25 years. Currently, every third octogenarian in the industrialized countries suffers from LOAD [1]. A much rarer disease variant referred to as early-onset AD (EOAD) develops at an earlier age (per definition < 65 years) and shows a more rapid clinical decline [2]. Both disease variants are characterized neuropathologically by extracellular senile plaques containing β-amyloid (Aβ) and intracellular neurofibrillary tangles comprising hyperphosphorylated tau protein [3]. Three proteins including amyloid precursor protein, presenilin 1 and presenilin 2 play an important role in the formation of Aβ [4]. However, mutations in these three proteins have been found only in EOAD but not in LOAD [5], which latter variant nonetheless, shows heritability between 60 to 80% in twin studies [6]. Nevertheless, genome-wide [7–13] and candidate-driven studies [14] demonstrated that several single-nucleotide polymorphisms (SNP) associate with the disease. Nevertheless, 20% of the genetic risk remains undetected [15] and completing a shortlist of LOAD-associated SNP candidates could further investigation on their potential roles in the etiology of LOAD.

Definite diagnosis of a disease status is crucial but this is only possible post mortem in LOAD. Thus, routinely, examination of probable LOAD is based on medical and familial history, exclusion of other causes, brain imaging techniques (CT, MRI, PET) and neuropsychological examination of cognitive functions [16, 17]. The latter are assessed by a wide variety of tests, e.g. the Mini Mental State Examination (MMSE) and the extended Consortium to Establish a Registry for Alzheimer's Disease test battery (CERAD-Plus). MMSE is a ten-minute screening test and comprises 30 questions that are totaled up into a single raw score [18]. In contrast, the detailed CERAD-Plus, which includes the MMSE component, takes approximately one hour. It lists z-values of fourteen subtests that are corrected for age, gender, and education [19]. Despite the limitations inherent to MMSE, it is nevertheless still used as the sole criterion for classification of cognitively healthy controls in many studies [8, 20–22]. The same neuropsychological tests are used for identification of the preceding stage of LOAD referred to as mild cognitive impairment (MCI), which is defined as “not normal, not demented” [23]. It represents the transitional phase between cognitive health and LOAD and, thus, is a heterogeneous group that includes converters to LOAD, reverters to normal cognition and stable MCI patients. Patients that are likely to progress to probable LOAD are mainly characterized by memory impairment [24].

The most important genetic marker for LOAD is the SNP pair allelic variant *APOE4* (rs429358, rs7412), translating into Arg112 and Arg158. Two other major alleles of *APOE* have been identified; with *E3* (Cys112, Arg158) being the most common and the *E2* variant (Cys112, Cys158) representing a rather rare but protective allele [25]. One copy of the *APOE4* allele confers a fourfold increased risk for LOAD, while two copies raise the risk tenfold [26]. Likewise, this allele is associated with a 1.4-fold increased risk for MCI [27].

A further SNP, which we recently found to associate with the *APOE4*-negative phenotype of LOAD [28], is rs4925 (GSTO1*C>*A*) and lies in the glutathione S-transferase omega-1 (*GSTO1)* gene. However, while we found a significant association of the major allele variant GSTO1*C, originally the minor variant GSTO1*A was shown to delay age-at-onset of LOAD [20, 29]. Nonetheless, association of the SNP with neither onset nor risk was confirmed by other studies [7, 8, 10–12, 30–33].

In the present study, we investigated whether the allele GSTO1*C could also serve as genetic risk marker for MCI, in addition to LOAD. The study participants were classified using established criteria, and two SNPs were genotyped by DNA amplifying techniques. Additive logistic regression results are discussed with reference to other association studies and commonly used classification strategies. A potentially negative impact on identifying significant, established and novel SNP-disease associations is illustrated by a comparison of the neuropsychological tests CERAD-Plus and MMSE.

## RESULTS

### The allele GSTO1*C (rs4925) is significantly associated with MCI

Previously, we showed that GSTO1*C significantly associated with the *APOE4*-negative LOAD phenotype [28]. Since MCI represents a transitional phase between cognitive health and LOAD, similarities in genetic characteristics to the latter might be expected. Hence, we investigated the prevalence of this allele in MCI.

We enrolled 52 MCI patients and 113 age- and sex-matched controls between the age of 65 and 95 years, who completed the CERAD-Plus test battery including MMSE and underwent clinical examination. MCI cases were diagnosed by a physician and a psychiatrist or psychologist according to the criteria of the consensus conference in Stockholm in 2003 [23] and the CERAD Diagnostic Manual for Dementia [34]. Neuropsychological criteria of MCI were an MMSE ≥ 24, not demented, intact activities of daily living, and impairment in at least two domains of memory with z ≤ −1.29 SD (CERAD) similar to the diagnostic comprehensive criteria [35]. The control test persons were classified as normal when MMSE ≥ 26 and, at most, one neuropsychological measure of the CERAD-Plus test battery fell more than 1.28 SD below age-appropriate norms (z ≤ −1.29 SD) in any cognitive domain [35]. Their MMSE values were in the range between 26 and 30, similar to the normative group for the German CERAD-Plus test battery [19, 36], see demographic overview, Table 1. Additionally, the study participants were genotyped for *APOE4* and rs4925 (*GSTO1*) using DNA amplification procedures. Both SNPs were consistent with Hardy-Weinberg equilibrium in both test groups.

**Table 1:** Demographic overview and rs4925 genotype characteristics of control and MCI sample donors

Samples	N	Mean age (SD, age range)	Gender Nf (%)	Mean MMSE (SD)	AF (*APOE4*)	N*E4/E4* (%)	rs4925 genotype, N		
							CC	CA	AA
**Controls**	113	79.1 (8.0, 65-95)	77 (68.1)	28.7 (1.2)	0.066	0 (0.0)	53	52	8
**MCI**	52	80.8 (6.5, 67-91)	41 (78.8)	27.4 (1.3)	0.173	2 (3.9)	33	18	1

Fifteen controls were heterozygous for *APOE4* (13.3%) and 14 MCI patients were heterozygous for *APOE4* (26.9%).

Abbreviations: N…number, SD… standard deviation, f… female, AF…allele frequency, E4/E4…*APOE4*/*APOE4* homozygous genotype

Logistic regression applied to the reference genetic marker *APOE4* yielded an OR 3.0 and a statistical significance *p = 0.005*. A logistic regression for GSTO1*C showed an OR 1.9 and *p* = 0.032 (Table 2).

Hence, GSTO1*C appeared to moderately associate with MCI. *APOE4* is also a published genetic marker for MCI [27] and, thus, confirmed the diagnosis procedure for the present study participant set. However, association of GSTO1*C with cognitive impairment was previously not observed [7, 8, 10–12, 30–33]. In order to identify potential reasons for these discrepancies, we compared the classification efficiencies of commonly used study designs and their impact on the significance of the associations between *APOE4* or GSTO1*C and MCI.

**Table 2:** Association (logistic regression analyses) of GSTO1*C or *APOE4* with MCI

SNP	B(SE)	Odds ratio (95% CI)	Sign.
rs429358 (*APOE4*)	1.084 (0.383)	3.0 (1.4 - 6.3)	0.005
rs4925 (GSTO1*C)	0.651 (0.304)	1.9 (1.1 - 3.5)	0.032

Binary logistic regression analyses comparing 113 controls and 52 MCI patients were performed on the GSTO1*C allele or the *APOE4* allele.

Abbreviations: B…Beta value, SE…standard error, CI… confidence interval, Sign. … significance

### The CERAD composite score outperforms MMSE in control/MCI classification

As indicated earlier, many LOAD biomarker studies have defined their control groups, partly or entirely, on the sole basis of MMSE, e.g. [8, 20–22]. However, it was reported that MMSE was a screening test for intermediate to severe LOAD [37] and had substantial false-negative rates [38]. Therefore, we examined, whether individual neuropsychological tests, especially MMSE, achieved the classification accuracy required for detecting significant associations of *APOE4* or GSTO1*C with cognitive impairment. First, classifications based on either MMSE or CERAD-Plus (without MMSE) were analyzed for their agreement with the group assignment determined by the standard consensus [23], CERAD [34] and comprehensive criteria [35], as described above. Afterward, the respective control and MCI classifications defined by either of the two neuropsychological tests were used to test whether the associations between *APOE4* or GSTO1*C and MCI observed in the previous section were retained.

The test persons described in the first section had been classified according to standardized consensus criteria [23, 34, 35], using information from clinical examination, medical history and neuropsychological testing. However, in order to ensure a tighter focus on the transition between cognitive health and MCI, the threshold MMSE ≥ 24 for MCI patients of the standardized criteria [23, 34, 35] was raised to MMSE ≥ 26. Consequently, 49 MCI patients and 113 age- and sex-matched controls were used for evaluation (Supplementary Table 1).

Logistic regression analyses were applied to analyze the degree of consistency between the classifications by MMSE or CERAD-Plus (without MMSE) and the standardized comprehensive criteria [23, 34, 35]. The logistic regression of the MMSE showed an accuracy of 74.1% and a significance *p* = 4.6 E-08 (Methods). The area under the curve (AUC) of the receiver operating curve (ROC) was 0.757, using a cut-off at 0.5 in the logistic regression analysis. The latter is equivalent to a cut-off MMSE ≤ 27 for classification of MCI. In contrast, a logistic regression of five CERAD subtests (backward likelihood ratio of 13 CERAD subtests excluding MMSE) calculated a prediction model for MCI with an accuracy of 89.5%, *p* = 2.1 E-25 and an AUC = 0.965 (cut-off 0.5). The calculated predicted probability for MCI for each individual can take values between 0 and 1 (>0.5 for MCI) and was termed “CERAD composite score”. Table 3 shows that the five CERAD subtests Z Wordlist total, Z WL savings, Z WL recognition, and Z Figure recall are part of this composite score. The statistical approach applied here was similar to the one used for computing a previous CERAD score for the identification of early LOAD [39].

Therefore, MMSE was only 83% as accurate as the CERAD composite score and it was important to determine whether the two test's increased error rates would nullify the association of *APOE4* and GSTO1*C with MCI.

**Table 3:** Association of CERAD (binary logistic regression analysis) with the classification of 113 controls and 49 MCI patients (CERAD composite score)

CERAD Subtest	B(SE)	Odds ratio (95% CI)	Sign.
Z verbal Fluency Animals	−0.644 (0.311)	0.5 (0.3 - 0.97)	0.038
Z Wordlist total	−2.222 (0.536)	0.1 (0.04 - 0.3)	3.4E-5
Z WL savings	−1.132 (0.359)	0.3 (0.2 - 0.7)	0.002
Z WL recognition	−0.790 (0.320)	0.5(0.2 - 0.9)	0.014
Z Figure recall	−1.125 (0.271)	0.3 (0.2 - 0.6)	3.2E-5

Stepwise logistic regression analysis (backward elimination, exclusion criterion: *p* ≥ 0.05; inclusion criterion: p < 0.05; cut-off value = 0.5) comparing 113 controls (MMSE ≥ 26) and 49 MCI patients (MMSE ≥ 26) was done on all 13 z -transformed CERAD subtests (excluding MMSE). Five subtests remained in the equation and performed much better (accuracy 89.5%) than the MMSE test (accuracy 74.1%); R^2^ = 0.632 (Hosmer&Lemeshow), R^2^ = 0.539 (Cox&Snell), R^2^ = 0.763 (Nagelkerke), Model chi-squared = 125.53 and *p* = 2.1 E-25). The resulting CERAD composite score (0 to 1) is represented by the probability P for diagnosis of MCI: P(MCI)=1/(1+e^(− (− 1.718 - 0.644 * Z verbal Fluency Animals - 2.222 * Z Wordlist total - 1.132 * Z WL savings - 0.790 * Z WL recognition - 1.125 * Z Figure recall))), P(MCI) > 0.5 implies MCI classification.

Abbreviations: B…Beta value, SE…standard error, CI… confidence interval, Sign. … significance

### MMSE fails to detect the associations of *APOE4* and GSTO1*C with MCI

Next, we investigated whether classification by either MMSE or the CERAD composite score would detect the association of *APOE4* and GSTO1*C with MCI. For this purpose, we used the group assignments obtained from the individual neuropsychological tests in the previous section. The respective group allocations were related to either *APOE4* or GSTO1*C as predictors, using logistic regression analyses.

The results showed an OR 1.6 and *p* = 0.255 for the association of the LOAD-specific *APOE4* variant with MMSE (Table 4, upper panel). On the other hand, the logistic regression of *APOE4* with the CERAD composite score revealed an OR 3.1 and *p* = 0.004 (Table 4, lower panel). Similar regression analyses of GSTO1*C exposed an OR 1.5 and *p* = 0.196 for MMSE (Table 4, upper panel), while the CERAD composite score showed an OR 1.9 and *p* = 0.045 (Table 4, lower panel).

These results demonstrated that classification by MMSE nullified the association of *APOE4* and GSTO1*C with MCI. Next, we looked into whether an association of GSTO1*C with LOAD would also be canceled when the use of MMSE for group assignment was restricted to the controls in a LOAD-control study.

**Table 4:** Association of GSTO1*C or *APOE4* with the MCI classification based either on MMSE or the CERAD composite score (49 MCI patients, 113 controls)

Test	SNP	B(SE)	Odds ratio (95% CI)	Sign.
MMSE	*APOE4* (rs429358)	0.437 (0.384)	1.6 (0.7 - 3.3)	0.255
	GSTO1*C (rs4925)	0.397 (0.307)	1.5 (0.8 - 2.7)	0.196
CERAD	*APOE4* (rs429358)	1.128 (0.386)	3.1 (1.5 - 6.6)	0.004
	GSTO1*C (rs4925)	0.636 (0.317)	1.9 (1.02 - 3.5)	0.045

Binary logistic regression analyses comparing controls and MCI patients that had been assigned either on the basis of the MMSE score (Methods) or the CERAD composite score (Table 3) were performed on the *APOE4* allele or the GSTO1*C allele (rs4925). The genetic markers *APOE4* and GSTO1*C are only significantly associated with the CERAD composite score-based classification (lower panel).

Abbreviations: B…Beta value, SE…standard error, CI… confidence interval, Sign. … significance

### Classifying controls by MMSE annulled the association of GSTO1*C with LOAD

MMSE alone is often used to classify healthy controls, applying a threshold of MMSE ≥ 26, e.g. [8, 20–22], which also covers the MMSE range for MCI patients. Therefore, we included a mathematical approach to analyze the association of GSTO1*C and *APOE4* with LOAD, in dependence of the classification procedure for the control group. For this purpose, a partially new study set comprising 69 cognitively healthy individuals (64 of which were identical with those in the previous sections) and 71 newly recruited LOAD patients within the age range of 65 to 95 years was genotyped for GSTO1*C and *APOE4* (Supplementary Tables 2 and 3). LOAD patients were diagnosed according to the Neurological and Communicative Disorders and Stroke and the Alzheimer's Disease and Related Disorders Association criteria (NINCDS-ADRDA) and controls were classified using the above standardized comprehensive criteria, [50] and [19, 33, 34]. Logistic regression would show whether GSTO1*C and *APOE4* were associated with the LOAD phenotype. Thereafter, our previous group of 49 MCI patients that was classified according to the standardized comprehensive criteria [19, 33, 34] and had an MMSE ≥ 26 was added to the group of healthy controls in this study, now totaling 118 individuals. The rationale behind this strategy was to determine by logistic regression whether the strength and significance of the original association between GSTO1*C or the published reference allele *APOE4* and LOAD would be nullified or reduced.

The results of a logistic regression of the original 69 controls and 71 LOAD patients revealed a significant association of GSTO1*C with the disease, OR 1.8 and *p = 0.045*. The values for association between the reference allele *APOE4* and LOAD were OR 6.7 and *p = 5.0 E-7* (Supplementary Table 3, Panels A1 and A2). Thereafter, 49 MCI patients with MMSE ≥ 26 were added to the 69 controls and logistic regression with the enlarged control group and 71 LOAD patients was performed. This resulted in the elimination of the originally significant association of GSTO1*C, OR 1.4, *p = 0.170*. The *APOE4* association values were reduced to OR 3.4 and the significance deteriorated to *p = 3.4 E-5* (Supplementary Table 3, Panels B1 and B2).

Hence, classification of the control group on the basis of an MMSE threshold seemed to cancel a significant association of GSTO1*C and LOAD, and additionally served to decrease the OR for the reference marker *APOE4* in LOAD. These findings are discussed below.

**Table 5:** Primers used for the *APOE* PCR and the ARMS PCR for rs4925

primer	Sequence	Tm (°C)	Conc. (μM)
APOE-FW	GACGCGGGACGGCTGTCCAAGGAGCTGCAGG CGACGCAGGCCCGGCTGGACGCGGACATGGA GGA	99.6	1.0
APOE-RV	AGGCCACGCTCGACGCCCTCGCGGGCCCCGGCCTGGTACACT	90.7	1.0
GSTO1-FWinnerA	TATTAGAAGCCAAAATAAAGAAGACTACGA	56	0.90
GSTO1-RVouter	GAAAGTGGGAATATAAAGAAAAGAATAGGA	56	0.60
GSTO1-FWouter	CGATACAGTTAGCCATAAACTGATAAACTAA	56	0.60
GSTO1-RVinnerC	ATTCTTTACGAAATTCTTCTTTTAGGCTAG	56	0.84

The resulting fragments for the *APOE* genotyping are explained in Methods. The ARMS PCR for rs4925 generates a total fragment of the two outer primers (239bp) that serves as a positive control, a major C allele-specific fragment (132bp) and/or a minor A allele-specific fragment (167bp). All sequences are given in the 5′ to 3′ direction.

Abbreviations: Tm… melting temperature, Conc. … concentration, μM… 10E-6 mol/L

## DISCUSSION

In the present study, we showed for the first time a significant association of GSTO1*C with mild cognitive impairment. This novel observation, however, could only be made following careful neuropsychological examinations undertaken using the standardized comprehensive criteria [23, 34, 35]. In addition, the strongest genetic marker for late onset Alzheimer's disease, *APOE4* [40], also associated with MCI, confirming that this group represented a preceding stage of LOAD. Moreover, GSTO1*C significantly associated with LOAD, but this relationship could only be exposed using established patient norms (NINCS-ADRDA) and strict control criteria not offered by the MMSE protocol. We compared the CERAD and MMSE neuropsychological tests and found the former to be superior in classifying participants for studying cognitive impairment. Indeed, patient and control classifications based on MMSE alone failed to expose the association between GSTO1*C and *APOE4* with MCI. Thus, we underscored on a genetic basis the inutility of applying MMSE alone to the characterization of healthy controls in studies of cognitive impairment patients.

In reference to our comparison between the neuropsychological tests, CERAD and MMSE, our study cautioned against the rather common use of MMSE as the sole classification criterion, especially for the identification of controls (MMSE ≥ 26). MMSE's limited accuracy (74.1%) with regard to distinguishing the cognitively healthy from MCI resulted in the failure to detect significant SNP/MCI and SNP/LOAD associations. The latter's shortcoming was caused by MCI cases falsely classified as controls. The MCI's resemblance to LOAD and their percentage in the whole control group determined the negative effect on the SNP/LOAD association. In contrast, CERAD performed much better in control/MCI classification, with an accuracy of 89.5%. As expected, especially the episodic memory-related CERAD subtests were significantly associated with MCI (Z Wordlist (WL) total, Z WL savings, Z WL recognition, Z Figure recall), suggesting that they were affected first during disease development [41].

However, our findings that GSTO1*C associated with an increased risk for MCI and LOAD are in contrast to other investigations, which only observed an association with age-at-onset of LOAD [29, 30] or did not find any relevance for LOAD [7, 8, 10–12, 31–33]. We speculate that these differing results were caused by several factors (discussed below), which altered group assignments across the studies.

It has been demonstrated previously that LOAD overlaps with several cerebrovascular pathologies such as regional cerebral hypoperfusion and cerebrovascular lesions [42]. Indeed, in a case/control study, the minor GSTO1*A/A genotype of rs4925 showed elevated frequency in vascular dementia (VaD) patients [43]. Thus, an increased VaD proportion in LOAD cases would attenuate the GSTO1*C prevalence and might conceal its association with LOAD.

To date, the majority of MCI or LOAD patients in case/control studies is classified on the basis of neuropsychological and/or brain imaging tests. In particular, there is a multitude of neuropsychological tests that quantify different cognitive functions, which certainly affects case- as well as control definitions. For instance, correlation between CDR and CERAD showed only medium effect sizes around r = −0.5 [44, 45]. As a consequence, associations of SNPs with MCI or LOAD may vary between case/control studies.

On the other hand, samples for the control group are sometimes drawn from younger, population-based cohorts and age-matching with cases is bypassed [8, 10, 29]. We estimated that, as a consequence, up to about 10% of the control group may show a LOAD-related genetic profile. This value was based on the assumption that approximately 35% of a 40 to 50-year old population in industrialized countries will reach the age of 85 [46] at which time roughly 30% would probably suffer from LOAD. This translates to a 10% risk for LOAD in all living 40 to 50-year olds. This confounding effect may be enhanced by an increased morbidity and mortality conferred by other SNP-associated diseases. For instance, *APOE4* elevates the risk for cardiovascular disease [47] and, thus, increases mortality. Therefore, the AF(*APOE4)* in an age-matched reference group is expected to be reduced as compared to younger population-based cohorts. Accordingly, the AF(*APOE4*) of the control group in the present study (0.065) was only half the AF (0.11) of the average Austrian population [48].

From the collected evidence presented here, GSTO1*C (rs4925) could turn out to be a genetic risk factor for impairment of cognition in the elderly. Moreover, our finding that MMSE alone was not accurate enough to assign participants to the cognitively healthy control group may foster refinement of case/control study designs.

## MATERIALS AND METHODS

### Ethics statement

Investigation has been conducted in accordance with the ethical standards and according to the Declaration of Helsinki and according to national and international guidelines and has been approved by the authors' institutional review board.

### Study participants

Study participants between 65 and 95 years were recruited from Greater Vienna (Austria) and were all of Caucasian origin. LOAD patients were enrolled in geriatric care, retirement and nursing homes. Control individuals and MCI patients were recruited from spouses of LOAD patients, in retirement homes and by word of mouth.

All study participants underwent clinical examination including a review of the medical history to confirm the absence of systemic disorders. Neuropsychological testing was performed using the German CERAD test battery [49], which included MMSE, on the day of blood sampling.

71 clinically suspected LOAD patients additionally underwent structural brain scanning using MRI to exclude other causes of cognitive impairment like stroke or tumors. Diagnoses of probable LOAD were established by a physician and a psychiatrist or psychologist according to the criteria by the US National Institute of Neurological and Communicative Disorders and Stroke and the Alzheimer's Disease and Related Disorders Association (NINCDS-ADRDA) [50], which are equivalent to the standardized CERAD criteria [51]. LOAD patients showed two or more severe deficits in cognition, [34], and age between 65 and 95 years. Further selection criteria were severe temporal lobe atrophy on MRI and exclusion of other forms of dementia (i.e. VaD).

52 MCI patients were diagnosed by a physician and a psychiatrist or psychologist according to the criteria of the consensus conference in Stockholm in 2003 [23] and the Diagnostic Manual for Dementia [34]. Neuropsychological criteria of MCI were an MMSE ≥ 24, not demented, intact activities of daily living, and impairment in at least two domains of memory with z≤ −1.29 (CERAD) using diagnostic comprehensive criteria [35].

118 control subjects were classified as normal by a physician and a psychiatrist or psychologist when MMSE ≥ 26 and, at most, one neuropsychological measure of the CERAD test battery fell greater than 1.28 SD below age-appropriate norms in any cognitive domain (Table 1, Supplementary Table 2). Their MMSE values were in the range between 26 and 30, similar to the normative group for the German CERAD-Plus test battery [19, 36].

The study was approved by the ethics commission of the city of Vienna, Austria, EK-04-070-0604 and EK09/219/1209. Each participant and/or legal guardian was advised of the purpose and procedures of the study and written informed consent was obtained prior to initiating the study in accordance with the principles of the Declaration of Helsinki.

### Genotyping

Genomic DNA from whole blood was isolated according to the manufacturer's protocols using either QIAamp DNA Mini Kit (Qiagen) or PerfectPure DNA Blood Kit (5Prime). *APOE4* (rs429358, rs7412) genotyping was done according to [52] modified by R. Crook. In brief, genomic DNA was amplified using the APOE-FW primer and the APOE-RV primer (Table 5), the kit sample buffer, 10% DMSO, 200 μM dNTPs, 15 - 40 ng genomic DNA and 0.25 units HotStarTaq Plus DNA polymerase (Qiagen). PCR conditions on a Mastercycler epgradient S (Eppendorf) were: 94°C for 5min, 45 cycles: 94°C for 20s, 66.5°C for 30s, 72°C for 35s, final extension: 72°C for 10min. After HhaI digestion (Fermentas), 3% TBE agarose gel electrophoresis (0.025 μl/ml Midori Green, NIPPON Genetics or 0.05 μl/ml peqGREEN, peqlab) and documentation (Bio-Visionsystem, peqlab), the fragment patterns were interpreted as follows: *APOE2/APOE2* 144bp, 96bp; *APOE3/APOE3* 144bp, 48bp; *APOE4/APOE4* 72bp, 48bp; *APOE2/APOE3* 144bp, 96bp, 48bp; *APOE3/APOE4* 144bp, 72bp, 48bp; *APOE2/APOE4* 144bp, 96bp, 72bp, 48bp. The SNP rs4925 was genotyped using Amplification-refractory mutation system PCR [53], which allowed the detection of both SNP variants in a single reaction tube. The PCR reaction volume was 10 μl, using the kit sample buffer, 15-40 ng genomic DNA, 200 μM dNTPs, four primers (Table 5) and 0.25 units HotStarTaq Plus DNA polymerase (Qiagen). After an initial denaturation step at 95°C for 10min, a touch down PCR was run (10 cycles: 95°C for 45s, 68°C for 45s, 72°C for 1min 30s; 10 cycles: 95°C for 45s, 58°C for 45s, 72°C for 1min 30s; 18 cycles: 95°C for 45s, 54°C for 45s, 72°C for 1min 30s; 9 cycles: 95°C for 35s, 52°C for 45s, 72°C for 1min; final extension:72°C for 10min). The PCR reaction was loaded on a 2.5% TBE agarose gel and analyzed as described above. The resulting PCR fragments corresponded to the positive control generated by the two outer primers (239bp), the major C allele (132bp) and the minor A allele (167bp).

### Statistics

The calculations of associations between GSTO1*C (rs4925) or *APOE4* (rs429358) and MCI are part of an exploratory study, for which calculation of the statistical power was omitted.** Further testing in a new sample cohort will be necessary to confirm our findings. In order to estimate the statistical power for the association of *APOE4* or GSTO1*C with LOAD, we used simulation of a binary logistic regression model based on our previously published data [28]. The power of the association of *APOE4* with LOAD was predicted to be 87% for 16 individuals per group and reached 100% for the participant numbers used in this study. The association of GSTO1*C with LOAD was also considered exploratory and therefore, its statistical power of 55% was accepted. [R] packages plyr [54] and HardyWeinberg [55] were used for calculations [56].

Pearson's chi-squared test was used to test for deviation from the Hardy-Weinberg equilibrium. Binary logistic regressions were used to analyze the association of the SNP of interest with the respective disease state.

Binary logistic regressions comparing 113 controls (MMSE ≥ 26) and 52 MCI patients (MMSE ≥ 24) were done on the GSTO1*C allele (rs4925) or the *APOE4* allele. Of these controls, 64 individuals were identical with those in the Control/LOAD analysis below. Next, both neuropsychological tests, MMSE and CERAD (without MMSE) were tested for their ability to correctly classify study individuals. In order to focus on the transition between cognitive health and MCI, MCI patients with MMSE ≥ 26 were selected, giving a total of 49 MCI patients. Firstly, a logistic regression comparing 113 controls (MMSE ≥ 26) and 49 MCI patients (MMSE ≥ 26) was performed on MMSE values. The results for the prediction model were: B(SE) = −0.781 (0.152); OR 0.5 (0.3-0.6); *p* = 7.94 E-07; accuracy = 74.1%; correlation coefficients: R^2^ = 0.175 (Hosmer&Lemeshow), R^2^ = 0.168 (Cox&Snell), R^2^ = 0.238 (Nagelkerke); model chi-squared = 29.89 and *p* = 4.6 E-08. MMSE-based MCI classification was calculated using a predicted probability cut-off value > 0.5, which corresponded to MMSE ≥ 28 as cut-off for the logistic regression. Secondly, a stepwise logistic regression analysis with backward elimination (exclusion criterion: *p* ≥ 0.05; inclusion criterion: *p* < 0.05; cut-off value = 0.5) comparing 113 controls (MMSE ≥ 26) and 49 MCI patients (MMSE ≥ 26) was done on all 13 z-transformed CERAD subtests (excluding MMSE). Five subtests remained in the equation and the value for the predicted probability for MCI was called “CERAD composite score” (for details see Table 3 and legend): accuracy = 89.5%; correlation coefficients: R^2^ = 0.632 (Hosmer&Lemeshow), R^2^ = 0.539 (Cox&Snell), R^2^ = 0.763 (Nagelkerke), model chi-squared = 125.53 and *p* = 2.1 E-25. CERAD composite score-based MCI classification was calculated using a predicted probability cut-off value > 0.5. Finally, it was tested whether GSTO1*C or *APOE4* would still be found to associate with MCI, when study individuals were classified on the basis of either the MMSE logistic regression or CERAD composite score. Again, binary logistic regression analyses were used with GSTO1*C or *APOE4* as predictor variable and the respective classification as outcome (Table 4). The calculations of associations between the SNPs and MCI are part of an exploratory analysis, which was not corrected for multiple testing (α = 0.05). Further testing in a new sample cohort will be necessary to confirm our findings. Furthermore, a logistic regression analysis was performed on GSTO1*C or *APOE4* with 69 controls and 71 LOAD patients. SPSS Statistics 22 (IBM) was used for these calculations.

## SUPPLEMENTARY MATERIAL TABLES


