# Supplementary material for: Careful neuropsychological testing reveals a novel genetic marker, GSTO1*C, linked to the pre-stage of Alzheimer's disease
Source: Oncotarget. 2016 Jun 1;7(26):39108–17. doi: 10.18632/oncotarget.9773 (PMC5129917; doi:10.18632/oncotarget.9773)
Supplement: Supplementary file 1 [file oncotarget-07-39108-s001.pdf]

Careful neuropsychological testing reveals a novel genetic marker, *GSTO1*C*, linked to the pre-stage of Alzheimer's disease

Supplementary Material

Supplementary Table 1: Sociodemographic overview of the sample set that was used for comparison of the efficacy of CERAD and MMSE.

Group	N[a]	Mean age (SD, age range)	Gender N_f, (%)	Mean MMSE (SD)	AF (*APOE4*)	N *E4/E4* (%)	rs4925 genotype, N		
							CC	CA	AA
**Controls**	113	79.1 (8.0, 65-95)	77 (68.1)	28.7 (1.2)	0.066	0 (0.0)	53	52	8
**MCI**	49	80.7 (6.7, 67-91)	38 (71.0)	27.5 (1.2)	0.184	2 (4.1)	30	18	1

Fifteen controls were heterozygous for *APOE4* (13.3%) and 14 MCI patients were heterozygous for *APOE4* (28.6%).

Abbreviations: N…number, SD…standard deviation, f…female, AF… allele frequency, E4/E4…*APOE4*/*APOE4* homozygous genotype

Supplementary Table 2: Sociodemographic overview of Control and LOAD study participants.

Group	N	Mean age (SD)	Gender, N_f (%)	Mean MMSE(SD)	AF (*APOE4*)	N *E4/E4* (%)
Controls[a]	69	82.5 (6.0)	47 (68.1)	28.5 (1.3)	0.065	0 (0.0)
LOAD	71	84.2(5.1)	49 (69.0)	14.5 (7.2)	0.296	5 (7.0)

a…64 control samples are identical with 64 of 113 controls used in Tables 1 to 4.
Abbreviations: N…number, SD…standard deviation, f…female, AF… allele frequency, E4/E4…*APOE4*/*APOE4* homozygous genotype

Supplementary Table 3: Association (logistic regression) of *GSTO1*C* and *APOE4* with LOAD.

Panel	Allele	Group	N	AF	Odds ratio (95% CI)	Sign.
A1	*GSTO1*C*	Controls	69	0.69	**1.8** (1.01 - 3.03)	**0.045**
		LOAD	71	0.80		
A2	*APOE4*	Controls	69	0.07	**6.7** (2.95 - 15.16)	**5.8 E-7**
		LOAD	71	0.30		
B1	*GSTO1*C*	Controls plus MCI	118	0.73	1.4 (0.86 - 2.36)	0.170
		LOAD	71	0.80		
B2	*APOE4*	Controls plus MCI	118	0.11	**3.4** (1.89 - 5.96)	**5.0 E-6**
		LOAD	71	0.30		

Nine controls were heterozygous for *APOE4* (13.0%) and 32 LOAD patients were heterozygous for *APOE4* (45.1%).

Logistic regression analyses, comparing controls and LOAD patients, were performed on *GSTO1*C* (panel A1) or *APOE4* (panel A2) and showed significant associations with the disease. In Panel B1 the significance for the association of *GSTO1*C* with LOAD was lost, when the control group was confounded with MCI cases (42%) due to the frequently used cut-off MMSE $\geq$ 26 as the only control group classification criterion. The association of *APOE4* remained significant but OR decreased considerably.

Abbreviations: N…number, AF… allele frequency, CI… confidence interval, Sign. … significance
